# Evaluation of the role of the cannabidiol system in an animal model of ischemia/reperfusion kidney injury

**DOI:** 10.5935/0103-507X.20150064

**Published:** 2015

**Authors:** Rodrigo Zon Soares, Francieli Vuolo, Dhébora Mozena Dall'Igna, Monique Michels, José Alexandre de Souza Crippa, Jaime Eduardo Cecílio Hallak, Antonio Waldo Zuardi, Felipe Dal-Pizzol

**Affiliations:** 1 Laboratory of Experimental Physiopathology, Graduate Program in Health Sciences, Health Sciences Unit, Universidade do Extremo Sul Catarinense - Criciúma (SC), Brazil.; 2 Department of Neuroscience and Behavior, Faculdade de Medicina de Ribeirão Preto, Universidade de São Paulo - São Paulo (SP), Brazil.

**Keywords:** Cannabidiol/therapeutic use, Receptors, cannabinoid, Ischemia/metabolism, Reperfusion injury/metabolism, Kidney/injuries, Inflammation

## Abstract

**Objective:**

This work aimed to investigate the effects of the administration of cannabidiol in a kidney ischemia/reperfusion animal model.

**Methods:**

Kidney injury was induced by 45 minutes of renal ischemia followed by reperfusion. Cannabidiol (5mg/kg) was administered immediately after reperfusion.

**Results:**

Ischemia/reperfusion increased the IL-1 and TNF levels, and these levels were attenuated by cannabidiol treatment. Additionally, cannabidiol was able to decrease lipid and protein oxidative damage, but not the nitrite/nitrate levels. Kidney injury after ischemia/reperfusion seemed to be independent of the cannabidiol receptor 1 and cannabidiol receptor 2 (CB1 and CB2) expression levels, as there was no significant increase in these receptors after reperfusion.

**Conclusion:**

The cannabidiol treatment had a protective effect against inflammation and oxidative damage in the kidney ischemia/reperfusion model. These effects seemed to be independent of CB1/CB2 receptor activation.

## Abstract (pt)

**Objetivo:**

Investigar os efeitos da administração de canabidiol em um modelo de isquemia/reperfusão renal em animais.

**Métodos:**

Foi induzida uma lesão renal, por meio de 45 minutos de isquemia renal seguida por reperfusão. Administrou-se canabidiol (5mg/kg) imediatamente após a reperfusão.

**Resultados:**

A isquemia/reperfusão aumentou os níveis de interleucina 1 e fator de necrose tumoral, o que foi atenuado pelo tratamento com canabidiol. Além disso, o canabidiol foi capaz de diminuir o dano oxidativo de lipídios e proteínas, mas não os níveis de nitrito/nitrato. A lesão renal após isquemia/reperfusão pareceu ser independente da expressão dos receptores canabidiol-1 e canabidiol-2, já que não houve aumento significante desses receptores após a reperfusão.

**Conclusão:**

O tratamento com canabidiol teve um efeito protetor contra a inflamação e o dano oxidativo em um modelo de isquemia/reperfusão renal. Esses efeitos parecem não ocorrer via ativação dos receptores canabidiol-1/canabidiol-2.

## INTRODUCTION

Acute renal failure (ARF) is a clinical condition characterized by acute deterioration of renal function.^(1)^ ARF has a high incidence and mortality rate, mainly in older individuals with chronic diseases and critically ill patients.^(2)^ One of the major causes of ARF is renal hypoperfusion, which includes a final common death pathway in tubular cells.^(1)^

During ischemia, an inflammatory response occurs, neutrophils infiltrate the kidney tissue and release various cytokines, and renal vasoconstriction is present.^(3)^ Additionally, tubular cell damage and loss of cellular polarization occurs,^(4)^ which impairs the active transport of sodium and water, and is followed by obstruction of the tubular lumen.^(4)^ Furthermore, hypoxanthine accumulation occurs and, after reperfusion, its metabolism generates reactive oxygen species (ROS) production.^(3)^ In this context, several studies have attempted to demonstrate the protective role of anti-inflammatory substances in ischemia/reperfusion (I/R).^(5-10)^

Cannabidiol (CBD) is a non-psychotropic compound of the *Cannabis sativa* plant. Several pharmacological effects of CBD are mediated by its interaction with the cannabinoid receptors (CB1 and CB2). Expression of CB1 occurs predominantly in the central nervous system (CNS), whereas CB2 is mainly expressed in immune cells. CBD was shown to be a potent anti-inflammatory agent, and it exerts its effects through the induction of T cell apoptosis, inhibition of cell proliferation, cytokine production suppression, and regulatory T cell induction.^(11-16)^ Additionally, it was demonstrated that CB1 receptor inhibition could decrease the release of inflammatory mediators and ROS, leading to decreased renal epithelial cell death.^(15)^

Given the large number of animal studies demonstrating the importance of inflammation in the development of ischemic acute renal failure, and the lack of therapeutic options in the clinical setting, there is good rationale to believe that new anti-inflammatory agents may be an important adjunctive treatment for ARF. Thus, we hypothesized that the CB2 receptor would be up-regulated in an ARF animal model and CBD administration would be able to decrease kidney damage.

## METHODS

Male adult Wistar rats were obtained from the *Universidade do Extremo Sul Catarinense* - UNESC (Criciúma, Brazil) breeding colony. They were housed five per cage with food and water available ad libitum, and they were maintained on a 12-hour light/dark cycle (lights on at 7:00 AM). All experimental procedures involving animals were performed in accordance with the NIH Guide for the Care and Use of Laboratory Animals, with the approval of the UNESC Ethics Committee, number 112/2012.

CBD (99.9% pure) was kindly provided by THC-Pharm (Frankfurt, Germany) and STI-Pharm (Brentwood, UK). CBD was suspended in 2% polyoxyethylenesorbitan monooleate (Tween 80). The solution was prepared immediately before use and was protected from light.

Thirty male Wistar rats, weighing between 350 - 400g, were divided into three equal groups: a control group/Sham (Group 1), a kidney ischemia/reperfusion group (Group 2) and a kidney ischemia/reperfusion plus CBD group (5mg/kg) (Group 3).

The animals were anesthetized with ketamine (80mg/kg) and xylazine (20mg/kg) by an intraperitoneal injection. Then, an incision was made in the abdominal wall, and the renal pedicle was dissected bilaterally. The pedicle was clamped bilaterally for 45 minutes in Groups 2 and 3. In Group 1, the pedicle was manipulated but not clamped. Immediately before unclamping, a single dose of CBD (5mg/kg)^(12)^ or the same volume of saline was infused through the aorta. Twenty-four hours later, the animals were killed by decapitation and the kidneys were extracted.

Renal tissue samples were homogenized (50mg/ml) in 0.5% of hexadecyltrimethylammonium bromide and centrifuged. The suspension was sonicated and an aliquot of the supernatant was mixed with a solution of 1.6mM TMB and 1mM H_2_O_2_ (hydrogen peroxide). The myeloperoxidase (MPO) activity was measured spectrophotometrically at 650nm at 37°C. The results were expressed as mU/mg protein. The MPO activity was evaluated as a neutrophil infiltration indicator.

### Thiobarbituric acid reactive species levels

Samples were homogenized and incubated in 60 mM Tris-HCl, pH 7.4 (0.1 mM DTPA) and 0.73% thiobarbituric acid for 60 min at 100°C. After the samples were cooled for 15 min at 5°C, they were centrifuged and their absorbance levels were measured at 535nm. The thiobarbituric acid reactive species (TBARS) levels were expressed as nmol of malondialdehyde (MDA) per milligram of protein (nmol.g^-1^).^(17)^

Homogenized samples were precipitated by the addition of hydrochloric acid and the proteins were dissolved by guanidine (6M). 2.4 - dinitrophenylhydrazine was added and the formed Schiff base was measured at 370nm. The results were expressed as nmol of carbonyls per milligram of protein carbonylation.^(18)^

Samples were mixed with a vanadyl chloride solution and Griess reagent. Then, thirty to forty minutes later the absorbance at 540nm was determined.^(19)^ A standard solution of sodium nitrate was serially diluted (1.6mM to 200mM), and the nitrite and nitrate (NOx) levels were expressed as mM/mg protein.

### CB1 and CB2 receptor expression

The CB1 and CB2 protein levels were measured by western blotting using specific antibodies. The samples were homogenized in Laemmli buffer (62.5mM Tris- HCl, pH 6.8, 1% [w/v] sodium dodecyl sulfate [SDS], 10% [v/v] glycerol) and equal amounts of protein (30mg) were fractionated by polyacrylamide gel electrophoresis - sodium dodecyl (SDS-PAGE). Then, they were electrotransferred to nitrocellulose membranes. The electrophoresis efficiency was verified by Ponceau S staining, and the membranes were blocked in Tris - buffered saline Tween (TTBS: 100mM Tris- HCl, pH 7.5, containing 0.9% NaCl and 0.1% Tween 20) with 5% albumin. The membranes were incubated overnight at 4°C with polyclonal rabbit anti-CB1 or CB2 (1:1,000). A secondary anti-rabbit IgG at a dilution of 1:10,000 was incubated with the membranes for 2 hours and, subsequently, the membranes were washed in TTBS and the immunoreactivity was detected by chemiluminescence using an amplified electrogenerated chemiluminescence signal. A densitometric analysis was performed with the Image J software v.1.34^®^. All of the results were expressed as the relative ratio between the blot and the immunocontent of β-actin.

### Cytokines levels

Tumor necrosis factor alpha (TNF-α) and interleukin-1 beta (IL-1β) concentrations were determined with commercial ELISA (Enzyme-Linked Immunosorbent Assay) kits on a microplate reader (Peprotech, Ribeirão Preto, Brazil).

### Statistical analyses

The results are expressed as the mean ± standard deviation. Differences between groups were determined by one-way analysis of variance, followed, when appropriate, by the Tukey's test. All of the statistical analyses were performed with the Statistical Package for Social Science (SPSS) version 21.0 (SPSS, Chicago, IL, USA). Differences were considered significant when p < 0.05.

## RESULTS

**Figure 1:**
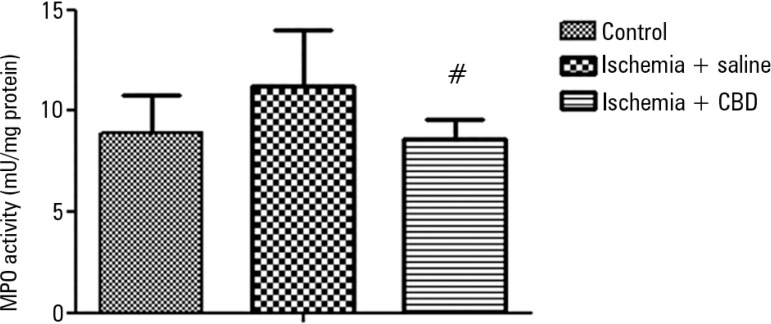
The effect of cannabidiol treatment on myeloperoxidase activity after renal ischemia reperfusion injury. Animals were sham operated or had their renal pedicle clamped bilaterally for 45 minutes. Immediately before unclamping, a single dose of cannabidiol (5mg/kg) or saline was administered. Twenty-four hours later, the myeloperoxidase activity was measured in the kidneys. MPO - myeloperoxidase; CBD - cannabidiol. Data are expressed as mU/mg protein. ^#^ Different from ischemia/saline. p < 0.05.

**Figure 2:**
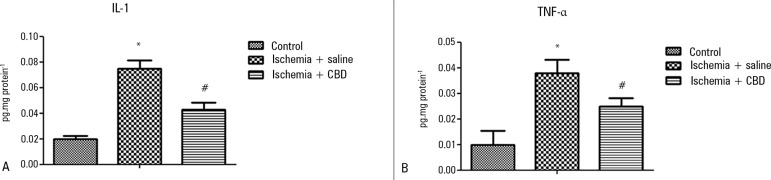
The effect of cannabidiol treatment on cytokine levels after renal ischemia reperfusion injury. Animals were sham operated or had their renal pedicle clamped bilaterally for 45 minutes. Immediately before unclamping, a single dose of cannabidiol (5mg/kg) or saline was administered. Twenty-four hours later, the IL-1 (A) and TNF-α (B) levels were measured in the kidneys. CBD - cannabidiol. Data are expressed as pg/mg protein. * Different from control; ^#^ different from ischemia/saline. p < 0.05.

Kidney inflammation was first investigated with MPO activity measurements. After I/R, there was a non-significant increase in the MPO activity in the kidney, and the CBD-treated animals presented a lower MPO activity when compared with the I/R animals [F = 4.15, p = 0.03] (Figure 1). In support of this effect, both the IL-1β and TNF-α levels were lower in the CBD-treated animals compared with the saline-treated animals [IL-1β: F = 548, p = 0.004; TNFα: F = 8, p = 0.001] (Figures 2A and 2B).

**Figure 3:**
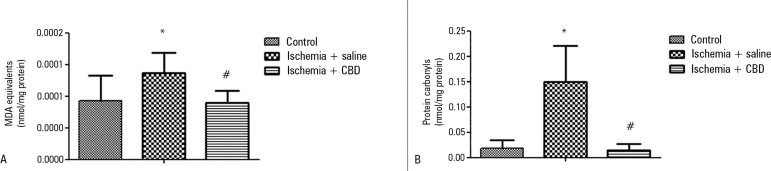
The effect of cannabidiol treatment on oxidative damage parameter levels after renal ischemia reperfusion injury. Animals were sham operated or had their renal pedicle clamped bilaterally for 45 minutes. Immediately before unclamping, a single dose of cannabidiol (5mg/kg) or saline was administered. Twenty-four hours later, the thiobarbituric acid reactive species (A) and protein carbonyls (B) levels were measured in the kidneys. MDA - malondialdehyde; CBD - cannabidiol. Data are expressed as nmol/mg protein. * Different from control; ^#^ different from ischemia/saline. p < 0.05.

**Figure 4:**
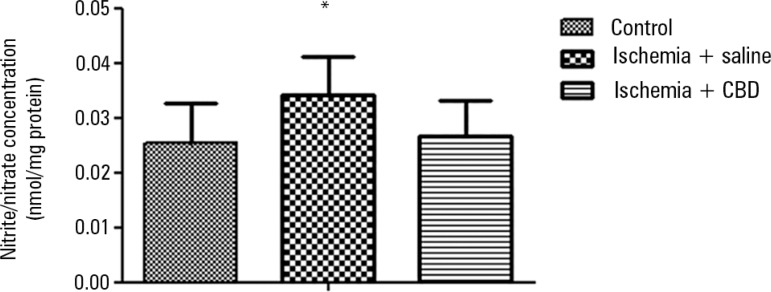
The effect of cannabidiol treatment on nitrite/nitrate levels after renal ischemia reperfusion injury. Animals were sham operated or had their renal pedicle clamped bilaterally for 45 minutes. Immediately before unclamping, a single dose of cannabidiol (5mg/kg) or saline was administered. Twenty-four hours later, the nitrite/nitrate levels were measured in the kidneys. CBD - cannabidiol. Data are expressed as pg/mg protein. * Different from control. p < 0.05.

As inflammation and oxidative damage are concomitant events, we evaluated oxidative damage to lipids and proteins, and both were increased after I/R (Figures 3A and 3B). Furthermore, we observed a decrease in these oxidative parameters in the CBD-treated animals [MDA: F = 6.56, p = 0.005; Carbonil: F = 20.57, p = 0.0001]. Another major mediator of oxidative stress and inflammation is nitric oxide (NO). The NO production was indirectly determined with the NOx quantification. As demonstrated with the oxidative damage parameters, there was a significant increase in the NOx levels after I/R. In contrast, the CBD-treated animals presented NOx levels that were similar to the sham-operated animals [F = 4.01, p = 0.03] (Figure 4).

**Figure 5:**
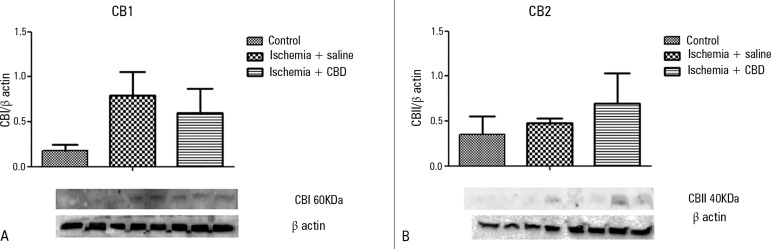
The effect of cannabidiol treatment on the CB1 and CB2 levels after renal ischemia reperfusion injury. Animals were sham operated or had their renal pedicle clamped bilaterally for 45 minutes. Immediately before unclamping, a single dose of cannabidiol (5mg/kg) or saline was administered. Twenty-four hours later, the CB1 (A) and CB2 (B) levels were measured in the kidneys. CBD - cannabidiol. The results were expressed as the relative ratio between the blot and the β-actin immunocontent. Order of bands: two bands Control; three bands Ischemia + saline; three bands Ischemia + CBD.

As CBD presented protective effects in this ARF animal model, we further characterized the expression of the cannabinoid receptors. I/R induction did not change the expression pattern of both CB1 and CB2 (Figures 5A and 5B). There was only a trend for higher CB1expression levels, which were not reversed by the CBD treatment [CB1: F = 2.09, p = 0.174; CB2: F = 3.93, p = 0.94].

## DISCUSSION

In the present study, we demonstrated that post-injury treatment with CBD was able to decrease kidney oxidative damage and inflammation in an animal model of ischemia-reperfusion. There was no significant variation in the CB1 and CB2 expression levels after reperfusion, suggesting that an up-regulation of CBD signaling did not occur in the development of kidney injury after I/R, although the therapeutic benefit of CBD remained.

MPO activity is proportional to the amount of kidney damage after I/R.^(20)^ Additionally, once damage occurs there, is an increase of several inflammatory mediators. This amplifies the initial inflammatory response and induces the expression of inducible nitric oxide synthase (iNOS), which provides a relevant role in the propagation of inflammation and oxidative damage.^(21-23)^ Since the cannabidiol system was first demonstrated in the central nervous system (CNS), there have been several descriptions of the anti-inflammatory effects of CBD in this context.^(24)^ The effects upon the CNS seem to be partially dependent upon adenosine receptors^(25)^ and CB2.^(26)^ Despite this, some of the anti-inflammatory effects of CBD seem to be dependent on its antioxidant effects.^(27)^ CBD affects genes that are classically associated with the regulation of stress responses, such as Nrf2, and this is in accordance with our results.

There is little information regarding the protective effects of CBD outside of the CNS. Moreover, even the peripheral protective effects of CBD could be mediated by the control of the neuroimmune axis.^(26)^ However, it was recently observed that CBD could actually worsen lung injury induced by lipopolysaccharide (LPS).^(28)^ Pre-treatment with CBD appeared to improve kidney function in an animal model of I/R.^(29)^ We demonstrate here, using a more clinically relevant model of post-injury administration, that CBD decreased kidney oxidative damage and inflammation in an animal model.

Unlike other cannabinoids, CBD has little affinity for the CB1 and CB2 receptors. The antioxidant and anti-inflammatory effects of this compound could have occurred through direct action or through some of the more recently characterized non-CB1/CB2 receptors.^(27)^ However, some evidence suggested that despite the low affinity for CB1 and CB2, CBD antagonizes CB1/CB2 receptor agonists, even at low concentrations.^(30)^ We demonstrated that the CB1 and CB2 receptors were not up-regulated after kidney I/R, suggesting that kidney injury mechanisms are not related to this pathway. These findings are in agreement with the idea that CBD does not act via the CB1/CB2 receptors, at least in the context of kidney injury.

We previously demonstrated that CBD exerts protective effects against LPS-induced lung injury, and this was partially regulated by the adenosine receptors.^(14)^ Furthermore, the anti-inflammatory actions of cannabinoid analogs, such as NAgly24 and ajulemic acid, have been attributed to their ability to promote the release of free arachidonic acid. In these examples, a result of this action was the formation of anti-inflammatory lipids, such as lipoxin A4 and 15d-PGJ2.^(31,32)^ A similar mechanism may explain some of the anti-inflammatory actions of CBD. The involvement of the A2A receptors has also been demonstrated, in which they can down regulate over-reactive immune cells, resulting in protection of tissues from collateral inflammatory damage. Additionally, it has been reported that CBD has the ability to enhance adenosine signaling through uptake inhibition and provide a non-cannabinoid receptor mechanism by which CBD can decrease inflammation.^(33,34)^ CBD treatment attenuated cisplatin-induced expression of NOX4 and NOX1 and the consequent renal oxidative stress. Additionally, CBD also decreased cisplatin-induced inflammatory responses, iNOS overexpression, and nitrotyrosine formation.^(35)^ The beneficial effects of CBD treatment in a mouse model of hepatic I/R injury were retained in CB2 knockout mice and were not reduced by CB1 or CB2 antagonists in vitro, suggesting an effect independent of receptor activation.^(15)^

Our results must be interpreted in light of some limitations. First, we did not measure kidney function markers; thus, we cannot ascertain if the anti-inflammatory effects will directly impact kidney function. Despite this, as kidney inflammation is closely related to dysfunction and the protective effects demonstrated here were robust, we do believe that this could be a minor limitation. Second, we did not present dose- and time-response curves, and because some authors demonstrated a detrimental effect of CBD,^(27)^ it is not possible to exclude a "U" shaped effect of CBD in our model.

## CONCLUSION

In conclusion, the present study suggests that cannabidiol treatment has a protective effect against inflammation and oxidative damage in the utilized kidney ischemia/reperfusion model. These effects seem to not be via CB1/CB2 receptor activation. Further studies targeting novel cannabinoid and other receptors may help to elucidate the exact mechanism of action by cannabidiol under inflammatory conditions outside of the central nervous system.
